# SMAD2 linker phosphorylation impacts overall survival, proliferation, TGFβ1-dependent gene expression and pluripotency-related proteins in NSCLC

**DOI:** 10.1038/s41416-025-02970-1

**Published:** 2025-05-03

**Authors:** Dörte Nitschkowski, Tim Vierbuchen, Holger Heine, Jochen Behrends, Norbert Reiling, Martin Reck, Klaus F. Rabe, Christian Kugler, Ole Ammerpohl, Daniel Drömann, Thomas Muley, Mark Kriegsmann, Georgious T. Stathopoulos, Kristina A. M. Arendt, Torsten Goldmann, Sebastian Marwitz

**Affiliations:** 1 Histology, Research Center Borstel - Leibniz Lung Center, Borstel, Germany; 2 Airway Research Center North (ARCN), Member of the German Center for Lung Research (DZL), Borstel, Lübeck and Großhansdorf, Germany; 3 Division of Innate Immunity, Research Center Borstel - Leibniz Lung Center, Borstel, Germany; 4 Core Facility Fluorescence Cytometry, Research Center Borstel - Leibniz Lung Center, Borstel, Germany; 5 Microbial Interface Biology, Research Center Borstel - Leibniz Lung Center, Borstel, Germany; 6 Department of Oncology, LungenClinic Grosshansdorf, Großhansdorf, Germany; 7 Department of Pneumology, LungenClinic Grosshansdorf, Großhansdorf, Germany; 8 Department of Thoracic Surgery, LungenClinic Grosshansdorf, Großhansdorf, Germany; 9 Institute of Human Genetics, University of Kiel, Kiel, Germany; 10 Medical Clinic III, University Medical Center Schleswig-Holstein (UKSH), Lübeck, Germany; 11 Translational Research Unit, Thoraxklinik at Heidelberg University Hospital, Heidelberg, Germany; 12 Translational Lung Research Center Heidelberg (TLRC-H), Member of the German Center for Lung Research (DZL), Heidelberg, Germany; 13 Institute of Pathology, University Hospital Heidelberg, Heidelberg, Germany; 14 Pathology Wiesbaden, Wiesbaden, Germany; 15 Helmholtz Center Munich, Molecular Carcinogenesis, Munich, Germany; 16 Comprehensive Pneumology Center Munich (CPC-M), Member of the German Center for Lung Research (DZL), Munich, Germany

**Keywords:** Non-small-cell lung cancer, Growth factor signalling

## Abstract

**Background:**

We investigated the impact of SMAD2 linker phosphorylation (pSMAD2L) on overall and disease-free survival, signal transduction, as well as cancer-related processes in non-small cell lung cancer (NSCLC).

**Methods:**

We generated A549 cells constitutively lacking pSMAD2L (A549L^sub^) to gain mechanistic insights and stimulated NSCLC cell lines with inhibitors against cell cycle-associated kinases or TGFβ1. In addition, we analysed SMAD2 and pSMAD2L in alveolar epithelial cells type 2 from tumour-free lung tissue as well as in benign and malignant T cells by Western blotting. Furthermore, pSMAD2L-positive tumours and immune cells were analysed in an NSCLC patient cohort (*n* = 316) using multiplex immunofluorescence.

**Results:**

In NSCLC cell lines and benign T cells, pSMAD2L was expressed in a mitosis-dependent manner. Loss of pSMAD2L (A549L^sub^) had an anti-proliferative effect, slowed migration, and increased alternatively spliced short SMAD2 (SMAD2^ΔE3^). The gene signature in A549L^sub^ was associated with developmental and morphogenetic processes and redirected canonical TGFβ1-dependent signalling. By contrast, SMAD2^ΔE3^ was absent in benign T cells but present in malignant T lymphoblasts. NSCLC patients with low pSMAD2L^+^ tumour cell density had a poorer prognosis, whereas low pSMAD2L^+^ immune cell density favoured overall and disease-free survival.

**Conclusions:**

pSMAD2L antagonises anti-proliferative canonical TGFβ-signalling in NSCLC and redirects TGFβ1-dependent gene expression, whereas loss of pSMAD2L enhances SMAD2^ΔE3^ and affects pluripotency-associated proteins in vitro. In NSCLC patients, pSMAD2L cell density influences disease-free and overall survival in a spatially distinct manner.

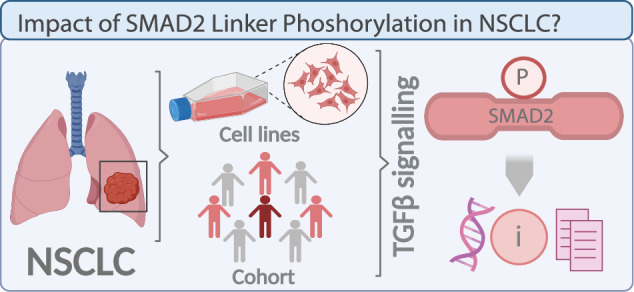

## Background

Lung cancer causes the highest cancer-related mortality worldwide [1, 2]. Histologically, 80%–85% of lung tumours are classified as non-small cell lung carcinoma (NSCLC), including the predominant subtypes lung adenocarcinoma (LUAD) and lung squamous cell carcinoma (LUSC) [3, 4]. Due to the absence of specific and early symptoms, the majority of patients are diagnosed at an advanced or metastatic stage, precluding curative surgical treatment [5]. In recent years, immunotherapies and targeted therapies have revolutionised therapeutic options and treatment modalities for advanced NSCLC by delaying cancer progression and extending patient survival. However, not all patients benefit from these novel therapies as they are neither eligible for immune checkpoint inhibitors nor exhibit addressable oncogenic aberrations. Thus, most lung cancer patients still receive conventional chemotherapy and radiation. In addition, NSCLC rapidly develops resistance to all available therapies, posing another major treatment challenge. Thus, more than 50% of patients still die within the first year of diagnosis, and the 5-year survival rate remains less than 18% [6, 7]. Therefore, elucidation of mechanisms underlying NSCLC is crucial for novel therapeutic strategies.

**Fig. 1:**
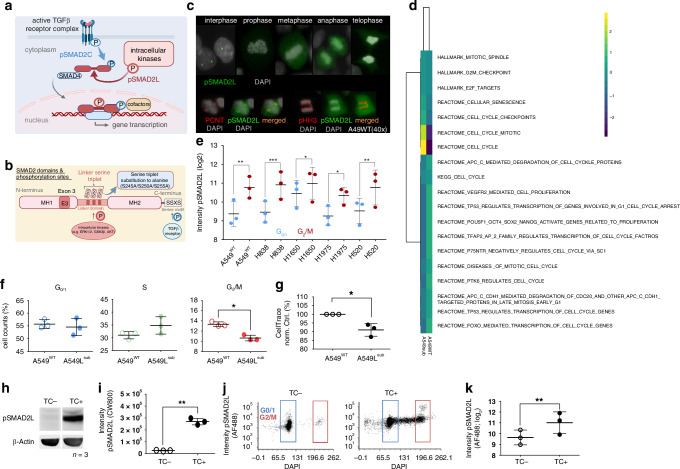
SMAD2 linker phosphorylation is associated with mitosis in NSCLC cell lines and benign T cells. **a** Schematic simplified illustration of the TGFβ signalling pathway in NSCLC cells. pSMAD2C is mediated by the activated TGFβ receptor complex (blue arrow), whereas SMAD2 linker phosphorylation (pSMAD2L) results from intracellular kinases (e.g., MAPK; red arrow). pSMAD2L/C translocates into the nucleus and drives TGFβ-dependent gene transcription in concert with SMAD4 and other cofactors (according to [15]). **b** Illustration of the SMAD2 linker-serine triplet, conserved C-terminal motif and mad homology (MH) 1, −2 domains. A549L^sub^ cells were generated by CRISPR/Cas9 carrying alanine instead of the serine triplet within the linker domain (S^245^A/S^250^A/S^255^A), preventing linker phosphorylation. **c** Expression of pSMAD2L in different cell cycle phases (top, A549^WT^ cells [*n* = 3]). Co-localisation of pSMAD2L with pericentrin (PCNT; bottom left) and phospho-Histone H3 (pHH3; bottom right) during mitosis in A549^WT^ cells (*n* = 3); magnification 40×. **d** Mitosis-related gene set collections were obtained from different platforms, and transcriptome data of A549^WT^ and A549L^sub^ (*n* = 3) were processed using the web-based Signature Visualization Tool (SaVanT). Cluster analysis of averaged z-scores for corresponding gene sets (see labels) is depicted. **e** Mean pSMAD2L fluorescence intensities (±SD; log_2_) of five different NSCLC cell lines in G0/1 and G2/M determined by flow cytometry. **f** Cell cycle analysis of A549^WT^ and A549L^sub^ cells performed by flow cytometry. **g** CellTrace™ proliferation assay is based on decreasing dye intensity per cell generation. Averaged normalised CellTrace™ fluorescence intensities (±SD) for A549L^sub^ and A549^WT^ are depicted, whereby the latter served as control (=100%). **h** Western blot result of pSMAD2L expression in activated proliferating T cells (TC^+^) and non-activated T cells (TC^−^) from healthy donors. β-Actin was used as a loading control. **i** Comparison of pSMAD2L abundance in TC^+^ and TC^−^ from healthy donors determined by Western blots (*n* = 3). **j** pSMAD2L intensities were analysed by flow cytometry with respect to cell cycle phases determined by DAPI in TC^+^ and TC^−^. **k** Overall pSMAD2L fluorescence intensities (log_2_) analysed by flow cytometry in TC^+^ and TC^−^ populations (*n* = 3); All pairwise comparisons were analysed by paired *t*-tests (*p* ≤ 0.05); *p ≤ 0.05; **p ≤ 0.01; ***p ≤ 0.001.

The transforming growth factor beta (TGFβ) signalling pathway is constitutively activated in NSCLC tissues and cell lines by epigenetic down-regulation of the pseudo-receptor bone morphogenetic protein and membrane-bound activin inhibitor (BAMBI), resulting in tumour growth, epithelial-mesenchymal-transition (EMT) and phosphorylation of the receptor-regulated small mother against decapentaplegic homologue 3 (SMAD3) [8]. SMAD2 and −3 (SMAD2/3) are key regulators and direct substrates of the TGFβ receptor complex that mediates C-terminal phosphorylation (pSMAD2/3C). Subsequently, pSMAD2/C translocates into the nucleus and acts as a transcription factor in concert with SMAD4 and other cofactors (Fig. 1a). In benign cells, pSMAD2/3C exerts an anti-proliferative effect, which is lost during cancer development. In the later stages of cancer, TGFβ serves as a key factor for carcinogenic EMT [9, 10]. Moreover, inhibition of TGFβ signalling in NSCLC by applying Pirfenidone in vitro and in vivo results in reduced proliferation, reduced viability of the tumours, and an increased immune cell influx, suggesting a possible role in therapy [11]. Furthermore, activation of the TGFβ pathway in immune cells of the tumour microenvironment (TME) has a negative impact on the overall survival of NSCLC patients [12]. However, regulation of SMAD2/3 is not restricted to receptor-mediated conserved C-terminal phosphorylation, as the proteins harbour additional phosphorylation sites in their divergent linker domain [13–15] (Fig. 1b). Thus, phospho-linker sites can be considered as interfaces for non-TGFβ pathway integration via intracellular kinases such as MAPK, protein kinase C, and c-Jun N-terminal kinase. Optional SMAD2/3 linker phosphorylation (pSMAD2/3L) leads to a variety of phospho-isoforms re-directing gene transcription and modulating proliferation, invasion, and cell growth, as discussed in the context of colorectal and hepatocellular carcinomas [13, 15–18]. Moreover, dual phosphorylation of pSMAD2/3 in the linker and C-terminal region (pSMAD2/3L+C) are observed in several cancers at the invasion front, while Smad2-deficient fibroblasts from mouse embryos (*Smad2*^−^^/^^−^ MEFs) expressing Smad2 mutants lacking linker serine/threonine (T^220^V/S^245^A/S^250^A/S^255^A) or C-terminal phosphorylation sites (S^464^A/S^465^A/S^467^A), as well as combinations of both, exhibit reduced infiltration capacity [13]. Therefore, Matsuzaki and colleagues hypothesised that preventing linker phosphorylation would shift malignant- to tumour-suppressive TGFβ signalling, providing a novel therapeutic approach for advanced cancers [13, 15]. In addition, SMAD2 plays a pivotal role during embryogenesis and is required for the maintenance of an undifferentiated pluripotent state in human embryonic stem cells and mouse epiblast stem cells [18, 19]. Taken together, these findings highlight that SMAD2 and phospho-isoforms are involved in fundamental cellular mechanisms and may represent a tunable phospho-interface for processes relevant in NSCLC.

Since TGFβ signalling is highly abundant in NSCLC [8], it represents a promising new target [11]. Therefore, our study aimed to investigate the presence and function of pSMAD2L, as NSCLC is still capable of proliferating despite an activated TGFβ signalling cascade, which requires processes to overcome the anti-proliferative effects of active TGFβ signalling. We hypothesise that linker phosphorylation of SMAD2 may be at least one way to achieve this in NSCLC.

## Materials and methods

### Cell culture, cell stimulation and cell line authentication

Cells were incubated at 37 °C and 5% CO_2_ in a humidified incubator and centrifuged at 300 × *g* for 5 min. Human adenocarcinoma (A549, H838, H1650, H1975) and squamous cell carcinoma (H520) cell lines were purchased from the American Type Culture Collection (ATCC) and JURKAT cells from the German Collection of Microorganisms and Cell Cultures GmbH (DSMZ, Braunschweig, Germany). Cells were maintained in RPMI-1640 supplemented with 10% FCS, 1% penicillin/streptomycin, and 1% stable glutamine (PAN Biotech, Aidenbach, Germany). For stimulation experiments, 2 × 10^6^–4 × 10^6^ NSCLC cells were seeded in T75 flasks (Corning GmbH, Kaiserslautern, Germany) with 10 ml RPMI to adhere overnight. Next day, medium was discarded and replaced with fresh media containing 10 µM Ro-3306 (CDK1 inhibitor), K03861 (CDK2 inhibitor), PD-0332991 (Palbociclib) (Selleck Chemicals GmbH, Planegg, Germany), 5 ng/ml human TGFβ1 (Thermo Fisher Scientific, PreproTech), or 1.5 mg/ml PIRF (TCI Chemicals GmbH, Eschborn, Germany). After incubation for 24- or 48 h, cells were harvested using trypsin/EDTA and the cell number was determined. Cell culture experiments were conducted simultaneously with a previously published study, and cell lines were authenticated by a certified service laboratory [11].

### Lung tissue and patient cohort

AECII were obtained from macroscopic tumour-free lung tissues. For mIF analyses, the NSCLC cohort comprised 316 NSCLC tissues (115 women, 201 men) with a median age at diagnosis of 65 years (interquartile range 58, 71), of which 186 were classified as LUAD and 130 as LUSC.

### Flow cytometry analysis

1 × 10^6^ cells were fixed for 15 min at 4 °C using 1% paraformaldehyde (PFA)-phosphate-buffered saline (PBS) solution in 5 ml tubes (Becton Dickinson GmbH, Heidelberg, Germany). Cells were resuspended with 3 ml PBS supplemented with 1% FCS (flow cytometry wash buffer, FCWB), followed by pelleting. The supernatant was discarded, and cells were permeabilized with 1 ml of pre-chilled methanol for 20 min at −20 °C. Cells were washed 3× with 3 ml FCWB. Finally, pellets were resuspended in 100 µl FCWB containing primary antibodies purchased from Cell Signaling Technology (Danvers, MA, USA): Rabbit anti-SMAD2 (SMAD2 total; clone D43B4, 1–500), rabbit anti-phospho-SMAD2 S465/467 (pSMAD2C; clone 138D4, 1–100), rabbit anti-phospho-SMAD2 S245/250/255 (pSMAD2L; 1–50), rabbit anti-SMAD3 (SMAD3 total, clone C67H9, 1–500) or rabbit anti-phospho-SMAD3 S423/425 (pSMAD3C; clone C25A9, 1–100) and mouse anti-phospho Histon H3 S10 (pHH3; clone 11D8, Alexa Flour®647 conjugated, 1–20; BioLegend, San Diego, CA, USA). After 60 min of incubation at RT, cells were washed and pelleted. Each sample received 100 µl of FCWB containing 2 µg of secondary antibody Goat anti-Rabbit Alexa Flour®488 (Thermo Fisher Scientific, Invitrogen, Waltham, MA, USA) and was incubated at RT for 45 min. Then, cells were washed 3× with 2 ml FCWB. As a negative control, cells were stained with the recommended isotypes corresponding to the highest primary antibody concentration. Then, 500 µl of a 3 µM 4′,6-diamidine-2-phenylindole, dihydrochloride (DAPI; BioLegend) solution was added to the cells and incubated for at least 15 min. Cells were analysed using the MACSQuant 10-Analyzer (Miltenyi Biotec B. V. & Co KG, Bergisch Gladbach, Germany). Laser voltages were maintained for all biological replicates. Data analysis was performed using FCS Express (V6) software (DeNovo, Pasadena, CA, USA). Gating strategies were adapted to the respective issues and included doublet exclusion. Data were then exported to MS Excel. Statistical analysis and visualisation were performed using GraphPad Prism (V6).

### Immunofluorescence microscopy (IFM)

24-well plates (Corning) were fitted with sterilised glass coverslips, and 1 × 10^5^ cells were seeded in 2 ml RPMI. After 24 h of incubation, cells were fixed with 2% PFA/PBS and permeabilized with 1 ml 0.25% Triton^TM^ X-100/PBS (Sigma Aldrich, St. Louis, MO, USA) for 10 min at RT. Coverslips were washed 3× with 2 ml FCWB and stained with rabbit anti-phospho-SMAD2 S245/250/255 (pSMAD2L; 1–50; Cell Signaling Technology) diluted in 500 µl FCWB and incubated for 60 min at RT. Cells were washed again as described and stained with 1 µg secondary antibody (goat anti-rabbit AF488; Thermo Fisher Scientific, Invitrogen) in 100 µl FCWB for each sample and incubated at RT for 45 min. If required, cells were simultaneously stained with mouse anti-phospho-Histone H3 serine 10 (pHH3; clone 11D8, 1–20; BioLegend) or mouse anti-pericentrin (PCNT) and TRITC secondary antibody (1–5000; Thermo Fisher Scientific). After washing, cells were covered with a 10 µM DAPI (BioLegend) solution for nuclei counterstaining and incubated for at least 15 min before being mounted upside down on glass slides using Fluoromount-G (Thermo Fisher Scientific, Invitrogen). Images from three different passages were obtained using the fluorescence Eclipse 80i microscope (Nikon, Tokyo, Japan) and DFC450 C camera (Leica, Wetzlar, Germany) with equal exposure times for each fluorochrome. Overlays were performed utilising the ImageJ (V1.53h) software.

### Protein extraction

Cells were lysed using Mammalian Protein Extraction Reagent™ supplemented with Halt Protease (1–100) and Halt Phosphatase (1–100) inhibitor (Thermo Fisher Scientific). Protein lysates were centrifuged at ≥8000 × *g* for 15 min. Supernatant was collected and protein concentration was determined using the BCA Assay Kit (Thermo Fisher Scientific) according to the manufacturer’s conditions. The readout was performed at 59 0 nm using a microplate reader (Tecan Group Ltd., Männedorf, Switzerland).

### Western blot

Samples were diluted with loading buffer under reducing conditions to a concentration of 25 µg per lane, followed by protein denaturation at 95 °C for 5 min. Gradient NuPAGE (4%–12%; Thermo Fisher Scientific) was used, and protein size was determined using pre-stained Chameleon Duo-Ladder (LI-COR Biosciences GmbH, Bad Homburg, Germany). Proteins were transferred to nitrocellulose membranes using the XCell SureLock® Mini-Cell system (Thermo Fisher Scientific, Invitrogen). Membranes were blocked for one hour using 1× Roti-Block (Carl Roth GmbH + Co. KG, Karlsruhe, Germany). For protein detection, primary antibodies (Cell Signaling technology) rabbit anti-SMAD2 (SMAD2 total; clone D43B4, 1–1000), rabbit anti-phospho-SMAD2 S465/467 (pSMAD2C; clone 138D4, 1–500), rabbit anti-phospho-SMAD2 S245/250/255 (pSMAD2L; 1–500), rabbit anti-SMAD3 (SMAD3 total; clone C67H9), rabbit anti-phospho-SMAD3 S423/425 (pSMAD3C; clone C25A9, 1–1000), rabbit anti-phospho-SMAD2 S245/250 (pSMAD2L_dual; clone 2H24L4, 1–500; Thermo Fisher Scientific, Invitrogen), rabbit anti-phospho-SMAD2 linker S250 (pSMAD2L_S250; clone EPR2855 [2], 1–500; Abcam, Cambridge, UK), or rabbit anti-phospho-SMAD2 linker S255 (pSMAD2L_S255; clone EPR2856(N), 1–500; Abcam) were diluted in 1× Roti-Block. Mouse anti-β-Actin (Abcam) served as a loading control. After one hour of incubation at RT, membranes were rinsed 3× with 1× tris-buffered saline Tween20 (TBST). For secondary antibody staining, IRDye 800CW Goat anti-Rabbit IgG (LI-COR) and IRDye 700CW Goat anti-Mouse IgG (LI-COR) were diluted in 1× Roti-Block (1–2000) and incubated for 45 min at RT, washed 3× with TBST, and finally rinsed with TBS before being analysed on the Odyssey Clx scanner (LI-COR). Protein intensities were analysed using the ImageStudio software (V4.0). Displayed images were adjusted in brightness and contrast for visualisation. Statistical analysis was performed using GraphPad Prism (V6).

### Primary human alveolar epithelial cells type 2 (AECII)

AECII extraction from tumour-free lung tissues was performed as previously described [20]. Protein lysates were analysed for SMAD2 total, pSMAD2L or pSMAD2C, and β-Actin (see “Western blot” section). The quantification of protein expression was determined using ImageStudio (V4.0) software, followed by statistical analysis using GraphPad Prism (V6).

### RNA extraction and integrity

Total RNA was extracted from ≤1 × 10^7^ cells using the RNeasy mini kit (Qiagen, Venlo, Netherlands) and eluted using 30 µl of RNase-free water. RNA concentration and quality were determined using the NanoDrop^TM^ 2000 (Agilent, Santa Clara, CA, USA). RNA quality was determined using the Bioanalyzer 2100 (Agilent) and the RNA 6000 Nano Kit (Agilent) according to the manufacturer’s conditions. Readout was performed using the Expert 2100 software, providing an RNA integrity number (RIN) depending on RNA quality. All RNAs used for gene expression analysis obtained RIN values ≥ 9.0 (out of 10.0). RNA samples were stored at −80 °C until use.

### Transcriptomes

Gene expression analysis was performed using 4 × 44k V2 Microarrays (Agilent) and the Low Input Quick Labeling Kit (Agilent) according to the manufacturer’s instructions as described [8]. The arrays were washed and analysed using the SureScan Microarray Scanner G2600D (Agilent). Signal extraction was performed using Feature Extraction (V11.5) software. Data were further processed using GeneSpring (V13), evaluating significantly differentially expressed genes using moderated *T*-test or ANOVA with Tukey’s post hoc Test or Benjamini–Hochberg (BH) multiple comparison correction (*p* ≤ 0.05). Gene Set Enrichment Analysis (GSEA) Molecular Signatures Database (MSigDB) was used for further analysis.

### SMAD2 linker-serine-triplet substitution

A549 cells bearing an alanine substitution in the SMAD2 linker triplet (S^245^A/S^250^A/S^255^A; A549L^sub^) were generated by homology-directed repair (HDR) using the clustered regularly spaced short palindromic repeats (CRISPR)/CRISPR-associated protein 9 (Cas9) system as described [21]. Single guide RNA (sgRNA) sequence 5′-GTAGTAGGAGATAGTTCTGC-3′ was used with the protospacer adjacent motif (PAM) 5′-TGG-3′. For cloning, we used plasmid PX458 (#48138, Addgene, Watertown, MA, USA) and the oligodeoxynucleotides (ODNs) SMAD2-1_fw: 5′ CACCGTAGTAGGAGATAGTTCTGC 3′ and SMAD2-1_rv: 5′ AAACGCAGAACTATCTATCTCCTACTAC 3′. The plasmid (PX458-SMAD2) was transformed into competent *E. coli* DH5α cells (New England Biolabs, Frankfurt am Main, Germany). For selection, Luria–Bertani agar plates supplemented with ampicillin were used. Plasmid isolation was performed using the HiPure Plasmid MiniPrep Kit (Thermo Fisher Scientific) according to the manufacturer’s instructions. HDR template SMAD2mut_fw: G•GAAATACTTCATTTATTTTTAAATCCTTTTTTAGGCGCTCCAGCAGAATTAGCTCCAACTACTCTTGCCTGTTAATCATAGCTTGGTAAGTTGCACATATGTTCC•C (Addgene) was obtained for substitution (phosphothioate bond (•)) containing *HaeII* restriction site. A549^WT^ cells (1 × 10^6^) were transfected with 1.5 µg PX458-SMAD2 and 2 µg SMAD2mut_fw HDR template using Cell Line Nucleofactor™ Kit V (Lonza, Basel, Switzerland). Cells were transferred into pre-warmed media and incubated at 37 °C for 48 h. Transfected cells were harvested, and GFP-positive cells were sorted by our Fluorescence Cytometry Core Facility at the Research Center Borstel using the FACSAria II cell sorter (Becton Dickinson). Single cells were expanded in 96-well plates for 21–30 days and fed with fresh media twice a week until PCR analysis (seqSMAD2-a_fw: 5′ CCATCAATGTGGATCGCGATG 3′ and seqSMAD2-a_rv: 5′ TGTGCCAGCAGAAAAGACTTAA 3′) was performed. The amplified 723 bp fragment was restricted by *HaeII* (New England Biolabs) for 30 min at 37 °C. Two fragments of 467 bp and 256 bp were expected when HDR was successful. DNA bands were detected by ethidium bromide and a 1-kb DNA ladder (New England Biolabs) in a 3% agarose gel exposed to ultraviolet light. We obtained two potential clones evaluated by sequencing (Eurofins Genomic, Ebersberg, Germany) using primer seqSMAD2-a_fw and seqSMAD2-a_rv. One cell clone exhibited the expected DNA base changes and was designated A549L^sub^ (Supplementary Fig. 2A, B). Cells were expanded and frozen (−80 °C) in stocks until use. The absence of SMAD2 linker phosphorylation was confirmed at the protein level by Western blot using four different antibodies against phospho-SMAD2 serine-linker sites (Supplementary Fig. 2C).

### Reverse transcriptase polymerase chain reaction (RT-PCR)

Complementary DNA (cDNA) was synthesised using the Maxima™ cDNA Synthesis Kit (Thermo Fisher Scientific) according to the manufacturer’s protocol. Exon3 (90 bp) spanning primers (S2_short_full_FWD: 5′ TGCCTTTGGTAAGAACATGTCG 3′; S2_short_full_REV: 5′ TGGAGACGACCATCAAGAGAC 3′) were used to differentiate between “full-length” and “short” SMAD2. DNA was extracted from the gel using the QIAquick Gel Extraction Kit (Qiagen) and sequenced by the National Reference Center (NRZ) for Mycobacteria at the Borstel Research Center. Transcript identities were analysed using the web-based NCBI Basic Local Alignment Search Tool Nucleotide (BLASTn) database.

### Multiplex immunofluorescence (mIF)

Multiplex staining of NSCLC tissues was performed using the Vectra® Polaris™ 1.0 scanner and Opal™ Polaris 7-Color Stain Kit (Akoya Biosciences, Menlo Park, CA, USA) according to the manufacturer’s instructions. FFPE tissue slides were de-paraffinized and rehydrated by xylene (2 × 10 min) and a graded series of alcohol (2× 100% 2 min, 90% 2 min, 80% 2 min, 70% 2 min) followed by washing in deionised water and TBST washing buffer. A cycle of mIF staining consisted of heat-induced antigen retrieval (HIER) with citric acid buffer pH 6 for 1 min at 1000 W and 10 min at 100 W using an inverter microwave (Panasonic) followed by protein block for 10 min using a blocking buffer (Akoya Biosciences). After blocking, the primary antibody was incubated for 45 min at room temperature, followed by 10 min of incubation with the OPAL HRP polymer. OPAL-TSA reaction was carried out for 10 min with the designated fluorochromes and dilutions. Between each step, rigorous washing was conducted using 1× TBST, and all incubations were carried out in a humidified dark chamber. All primary antibodies were diluted using antibody diluent (Zytomed Systems, Berlin, Germany). Endogenous peroxidases were quenched using 3% H_2_O_2_ after the first round of HIER. The tissue area was encircled with a PAP pen to ensure coverage with liquid. After the last cycle of mIF staining, the remaining polymer and antibodies were stripped off by incubation in citric acid pH 6 for 1 min at 1000 W and 5 min at 100 W. Nuclei were stained using spectral DAPI for 5 min. followed by washing with deionised water and mounting with coverslips using Prolong Gold antifade medium (Thermo Fisher Scientific).

For mIF staining, the following antibodies were used at designated positions of the mIF cycle, primary antibody dilution and OPAL-TSA combinations: 1. panCK (clone AE1/AE3, 1–300, Zytomed Systems, Berlin, Germany; OPAL 690, 1–150, Akoya Biosciences), 2. CD45 (clone D9M8I, 1–800, Cell Signaling Technology; OPAL 570, 1–150, Akoya Biosciences), 3. pSMAD2L (polyclonal, 1–100, Cell Signaling Technology; OPAL 620 1–100, Akoya Biosceinces), 4. pHH3 (clone 11D8, 1–800, Biolegend; OPAL 520, 1–300, Akoya Biosciences).

#### mIF imaging and data analysis

Whole-slide images of TMAs were acquired on a PhenoImager HT (Akoya Biosciences) at 20× magnification. Phenochart 1.1 (Akoya Biosciences) software was used to de-array the TMA image and set regions of interest for training of the image analysis algorithm and downstream batch analysis using inForm software 2.4 (Akoya Biosciences). For this, representative regions were selected to train a tissue segmentation algorithm to differentiate between stroma (consisting of immune and non-immune cells), tumour, as well as empty glass based on mIF markers such as panCK, CD45, as well as morphological characteristics. The tissue segmentation algorithm was tested on a subset of images and retrained on samples where the initial results did not reach sufficient quality. After tissue segmentation, individual cells were identified based on nuclear DAPI signal and additional information from mIF targets (panCK, CD45). Cell classification was conducted in a binary approach for each mIF target to identify mIF marker positive cells (i.e., CD45^+^) from negative cells for each employed mIF target. Batch analyses were conducted with an algorithm for each mIF marker across all TMAs. Samples with insufficient tissue segmentation results were excluded from downstream analyses, and the results from each algorithm were subsequently consolidated into one single dataset using the R package phenoptr 0.2.4 (Akoya Biosciences). The R package phenoptr reports 0.2.5. was used to extract cell density data (cells/mm^2^) of combined mIF cell classifier results for each region of tissue segmentation results (stroma/tumour). As all TMAs harboured 2–4 individual punches per patient, mean values per patient were calculated using the R package tidyverse 2.0.0 and plotted using the ggpubr 0.6.0R package.

#### Survival analysis

The survminer R package 0.4.9 was used for the survival analysis of cell density data from mIF analyses. The R package Boruta 8.0.0. was used to identify experimental variables impacting OS and DFS. For dichotomisation of continuous variables (cell density data) into high and low categories, the *surv_cutpoint* function was used based on the selected rank statistics [22] from the maxstat R package.

### Extraction and expansion of T cells

Heparinized whole blood samples from healthy donors were used for T cell extraction with the DYNAL™ Dynabeads™ Untouched Kit for Human T cells (Thermo Fisher Scientific) according to the manufacturer’s instructions. Extracted T cell populations were divided into (i) activation with T cell Expander CD3/CD28 Double Beads (Thermo Fisher Scientific, Gibco)+ IL2 (20 U/ml) (Thermo Fisher Scientific, PeproTech) and (ii) controls stimulated with solvents. After three days, detached beads were removed using a magnetic rack. 2 × 10^6^ T cells were fixed by 1% PFA/PBS and stained for purity using anti-CD3 and anti-CD45 antibodies (BioLegend) and analysed by flow cytometry. CD3/CD45 double positivity exceeded 93% in all biological replicates. Flow cytometry was performed using the MACSQuant 10-Analyzer (Miltenyi Biotec B. V. & Co KG, Bergisch Gladbach, Germany) and FCS Express (V7) software. Then, 1 × 10^6^ cells were re-seeded for a further three days and harvested for protein extraction and Western blot analysis. Statistics were performed with GraphPad Prism (V6).

### Cell proliferation assay

2 × 10^6^ cells with similar numbers of passages were stained after harvesting using CellTrace™ Far Red Proliferation Kit® (Thermo Fisher Scientific), following the manufacturer’s instructions. Subsequently, 1 × 10^6^ cells were directly fixed after staining with 1% PFA/PBS solution for 15 min and stored at 4 °C until further processing, serving as a starting reference. The other half of stained cells was re-seeded in T75 flasks containing 30 ml medium for a further 48 h before being harvested and fixed as well. Cells were permeabilized with pre-cooled methanol, stained with 3 µM DAPI and analysed using MACSQuant 10-Analyzer (Miltenyi Biotec B. V. & Co KG, Bergisch Gladbach, Germany). To compare proliferation rates, median intensities of starting references were compared with the corresponding final values after 48 h. For statistical analysis, GraphPad Prism (V6) was used.

### Wound healing assay

Cells were seeded in medium using 2-Well for Self-Insertion culture inserts (ibidi, Gräfelfing, Germany) at a density of 5 × 10^4^ per well and incubated overnight to adhere. The next day, the inserts were removed, and 1.5 ml of fresh medium was added, followed by 24 h of incubation. Cells were fixed with 1% PFA/PBS solution for 15 min at RT, permeabilized with pre-chilled methanol (−20 °C) and stained with 3 µM DAPI solution. Images were obtained using a fluorescence microscope (Nikon) equipped with a camera capable of capturing images at 4× magnification (Leica). The cell-free area was analysed using the ImageJ (V1.53h) MRI Wound Healing Tool plugin. Statistical comparison was performed using GraphPad Prism (V6).

### Cell invasion assay

Transwell® cell culture inserts with a pore size of 8 µm (12-well format; Coning) were coated with 500 µl Matrigel® Matrix Growth Factor Reduced (210 µg/ml; Corning) according to the manufacturer’s instructions. After gel polymerisation, 2 × 10^6^ cells previously deprived of FCS for 8 h were seeded in 500 µl medium into the coated apical well. The basal well was filled with 2 ml medium and incubated for 16 h. The next day, Matrigel was recovered using Matrigel® Recovery Solution (Corning). Inserts were washed three times in PBS before cells were detached from the upper and lower sides of the membrane using a trypsin/EDTA solution for 15 min. Cell suspensions from apical and basal wells were transferred into separate 5 ml tubes and washed 3× before being processed for flow cytometry analysis as described above. Calculated mean percentages of invasive cells were compared using GraphPad Prism (V6).

### Colony formation assay

6-well plates (Corning) were covered with 1.5 ml medium containing 0.6% UltraPure™ Low Melting Point Agarose (Thermo Fisher Scientific, Invitrogen). After polymerisation, harvested cells were diluted in 2.5 ml of medium and mixed with the same volume of a 0.6% agarose/medium solution (0.3%). Then, 1.5 ml with a final cell volume of 2 500 cells/ml was pipetted into each well onto the bottom layer. After polymerisation, plates were incubated for 21 days. Cells were fed twice a week with 300 µl fresh medium until colonies were counted. Three technical replicates were performed for each cell line in each assay. Colonies were stained with crystal violet. For each well, five fields of view from similar well regions were imaged for colony analysis using the Colony Count option embedded in the ImageJ (V1.53h) software. Averaged values were analysed for significance using GraphPad Prism (V6).

### MTT Assay

Metabolic activity was analysed using the MTT assay according to the manufacturer’s conditions (Merck, Darmstadt, Germany). Cells were cultured and seeded (1 × 10^4^) in phenol-free RPMI in 96-well plates and incubated for 24 h. The readout was performed using a plate reader (Magellan) at 570 nm. Statistical comparison and visualisation were performed with GrapPadPrism (V6).

### Protein Array

Proteome Profiler Human XL Oncology Array and Human Pluripotent Stem Cell Array (RnD Systems, Minneapolis, MN, USA) were used to compare protein abundances in cell lysates. Total protein concentration was determined by BCA, and 100 µg was applied per array. Immobilised antibodies were spotted in duplicate on nitrocellulose, including internal reference and negative control spots. Upon incubation, proteins bound to the membrane were labelled using a primary antibody cocktail followed by IRDye 800CW streptavidin staining (LI-COR). Detection was performed using the ODYSSEY Clx scanner (LI-COR), and fluorescence intensities were analysed using ImageStudio (V4) software. Averaged intensity values from negative controls were subtracted from specific spot signals before the means of biological replicates were calculated and tested for significance using GraphPad Prism (V6).

### Illustrations

Graphic illustrations were created using *BioRender.com* (graphical abstract, Fig. 1a, b; Fig. 5f, g; Fig. 7e).

## Results

### SMAD2 linker phosphorylation is expressed at elevated levels during mitosis and co-expressed with core molecules of cell division and proliferation

Phosphorylation of R-SMADs at the C-terminus is a common event in NSCLC tissues (Fig. 1a, b), and lung cancer continues to grow and disseminate, although C-terminal phosphorylation is known to exert anti-proliferative effects [8, 13]. We hypothesise that NSCLC is able to evade anti-proliferative effects mediated by pSMAD2/3C via non-TGFβ signaling pathways. Since TGFβ controls cell division [23] and phosphorylation of SMAD2 linker-serine 255 is increased during mitosis in HeLa cells [24], we first analysed various NSCLC cell lines to assess pSMAD2L expression in different cell cycle phases. Overall, A549 wild-type (A549^WT^) cells showed higher expression of pSMAD2L in mitotic cells, whereas cells in interphase exhibited few distinct spots, interestingly resembling centrosomes. Cells within prophase showed higher pSMAD2L levels, which were most pronounced during metaphase and anaphase (Fig. 1c, top). In addition, pSMAD2L was co-localised with phospho-Histone H3 (pHH3) and pericentrin (PCNT), highlighting the presence of pSMAD2L during mitosis in NSCLC in different cellular structures crucial to core cellular functions (Fig. 1c, bottom).

As pSMAD2L appears to play a central role in mitosis, we aimed to further investigate cellular consequences of SMAD2 linker phosphorylation by loss-of-function experiments. For this, we established an A549 cell line by substituting the coding sequences of serine to alanine in the SMAD2 linker region by CRISPR/Cas9 (S^245^A/S^250^A/S^255^A; A549L^sub^), thereby preventing SMAD2 linker phosphorylation via intracellular kinases (Fig. 1b). Successful cloning was verified by PCR and sequencing (Supplementary Fig. 1A, B). Western blot analyses using four different antibodies targeting different SMAD2 linker phospho-sites confirmed decreased abundances in A549L^sub^ at the protein level (Supplementary Fig. 1C). Transcriptome analysis was conducted to analyse differentially expressed genes of A549L^sub^ and A549^WT^ with respect to cell cycle and mitosis-associated gene sets from KEGG, REACTOME and MSigDB HALLMARK gene sets. Cluster analysis of gene collections showed that impaired pSMAD2L signalling affected mitosis-associated gene expression, with most pronounced differences for REACTOME_CELL_CYCLE_MITOTIC and REACTOME_CELL_CYCLE gene sets (Fig. 1d). Comparison of pSMAD2L expression in G0/1 and G2/M subpopulations of five different NSCLC cell lines revealed consistent results for all cell lines with significantly higher pSMAD2L abundance when cells entered G2/M phase compared with cells in G0/1 phase, confirming our previous findings (Fig. 1e). Additional cell cycle analysis using flow cytometry was performed in order to analyse the effect of missing SMAD2L phosphorylation on a functional level. A549L^sub^ exhibited a reduced number of cells within the G2/M phase compared to A549^WT^ (Fig. 1f). This was further validated by dye-dilution experiments showing a reduced proliferation within A549L^sub^ cell line compared to A549^WT^ (Fig. 1g). Overall, our results show that linker phosphorylation of SMAD2 is dynamically expressed during mitosis, and the absence of pSMAD2L results in decreased proliferation.

### SMAD2 linker phosphorylation during mitosis is a conserved phenomenon across different germ lines and a feature of both malignant and benign cells

To examine whether SMAD2 linker phosphorylation during mitosis is a feature unique to malignant NSCLC cells or a generic phenomenon, we analysed T cells from healthy donors as a model for non-malignant cells, which can be induced to proliferate by CD3/CD28 double-bead activation and IL2 stimulation in vitro. This also provided us with the opportunity to validate our results across germ lines, as respiratory epithelial cells arise from the endoderm and immune cells from the mesoderm during embryogenesis [25, 26]. Cell cycle analysis revealed a significant decrease in cells in G0/1 and an increase in subpopulations in G2/M and S of activated T cells, indicating successful induction of T cell proliferation (Supplementary Fig. 1D). Subsequently, we performed Western blot and flow cytometry analyses on activated and non-activated T cells. Interestingly, expression of total SMAD2 and pSMAD2C was increased in proliferating T cells (Supplementary Fig. 1E–G), whereas pSMAD2L was absent in non-proliferating T cells but expressed in activated cell populations (Fig. 1h, i). Elevated pSMAD2L levels were also associated with cells located in G2/M analysed by flow cytometry (Fig. 1j, k). Thus, our findings demonstrate that pSMAD2L expression during mitosis occurs in a germ-line spanning manner and appears to play a role in healthy and malignant cells, indicating a conserved role in key cellular processes. Therefore, we investigated which cell cycle-associated kinases mediate phosphorylation of SMAD2L and might represent actionable targets.

### CDK2 and CDK4/6 are responsible for SMAD2 linker phosphorylation in NSCLC

Since cyclin/CDK complexes are responsible for cell cycle progression, we stimulated five NSCLC cell lines with selective inhibitors against CDK1, CDK2, and CDK4/6 to identify which kinases mediate SMAD2L phosphorylation [27]. Cell cycle analyses and flow cytometry staining for pHH3 were performed to assess efficacy (Supplementary Fig. 2A–C).

**Fig. 2:**
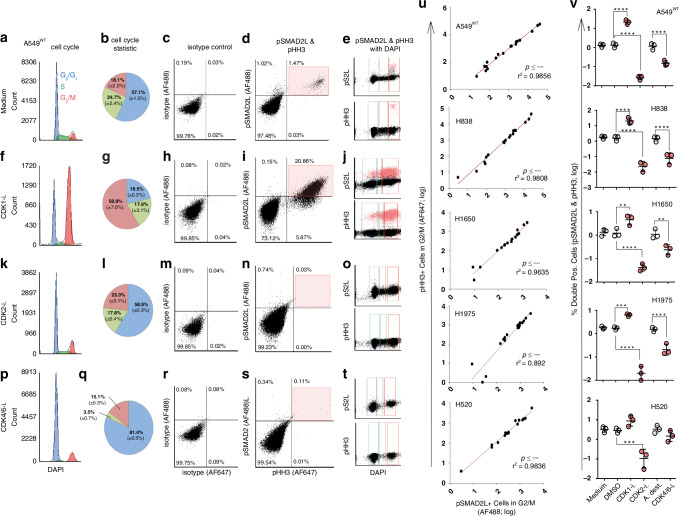
Effects of CDK inhibitors on cell cycle, pSMAD2L and pHH3 expression in NSCLC cell lines. NSCLC cell lines were stimulated with 10 µM CDK1, CDK2 and CDK4/6 inhibitors (CDK1-i.; CDK2-i.; CDK4/6-i.) or cultured in medium for 24 h and analysed by flow cytometry. Results of respective inhibitor controls are shown in the supplement (Supplementary Fig. 2D–M). **a**–**t** Flow cytometry analysis of A549^WT^ cells simultaneously stained for pSMAD2L (AF488) and pHH3 (AF647). For cell cycle analysis, DNA was additionally stained with DAPI. **u** pSMAD2L- and pHH3-positive cells (intensities ≥4 × 10^3^) in G2/M following CDK1-i., CDK2-i. and CDK4/6-i. Treatment and controls of three biological replicates (*n* = 18) of each cell line were subjected to Pearson correlation (*p* ≤ 0.01; two-sided). **v** Mean relative amounts of double positive pSMAD2L and pHH3 cells (%) were compared by RM one-way ANOVA (*p* ≤ 0.05); ****p ≤ 0.0001.

As expected, cells were arrested in G2/M when stimulated with the CDK1 inhibitor compared to medium, as CDK1 interacts with cyclin B in the late G2 phase, forming the mitosis-promoting factor [27] (Fig. 2a, b, f, g; Supplementary Fig. 2A, C). Similarly, pHH3+ cells were increased in G2/M after CDK1 inhibition compared to controls, and pSMAD2L-positive (pSMAD2L+) cells increased to a similar extent in G2/M (Fig. 2c–e and h–j; Supplementary Fig. 2D–M). We found that 92.8% (±6.9%) of pHH3^+^ cells in the G2/M phase were also positive for pSMAD2L among all NSCLC cell lines under medium conditions (*n* = 15; Fig. 2d).

CDK2 and CDK4/6 interact with cyclin A, D, and E in G1 and S phase to initiate and coordinate DNA replication [27, 28]. Consistent with this, we observed decreased subpopulations in the S phase when CDK2 and CDK4/6 inhibitors were applied. The effect was even more pronounced when the CDK4/6 inhibitor was used, which also resulted in G0/1 cell arrest (Fig. 2k, l and o, q; Supplementary Fig. 2A–C). However, stimulation of NSCLC cell lines with CDK2 or CDK4/6 inhibitors resulted in decreased amounts of pSMAD2L^+^ and pHH3^+^ cells in G2/M (Fig. 2m–o and r–t) and was validated by analysing double positive cell counts in different cell lines, independent of cell cycle phases. Correlation analysis of pSMAD2L^+^ and pHH3^+^ cell counts in G2/M under control and stimulation conditions resulted in a highly positive significant association of both markers, with a mean r-squared value of 0.9611 (±0.035) for all five cell lines (Fig. 2u, v). Taken together, CDK2 and CDK4/6, but not CDK1, are responsible for SMAD2 linker phosphorylation. Furthermore, both CDK2 and CDK4/6 activities are required to control SMAD2 linker and histone H3 phosphorylation adequately during mitosis. In order to complement flow cytometry data and to screen for possible splicing variants [29, 30], we performed Western blot analyses.

### Absence of pSMAD2L and inhibition of CDK4/6 lead to an increase of SMAD2^ΔE3^

**Fig. 3:**
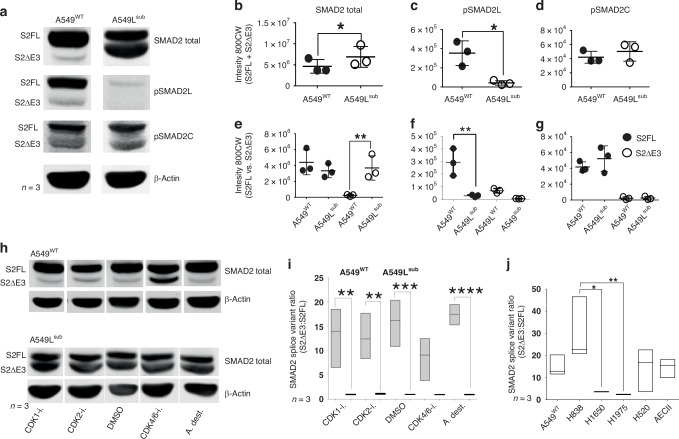
Expression of SMAD2 and phospho-isoforms in NSCLC cell lines, AECII and benign T cells. **a** Western blot of unstimulated A549^WT^ and A549L^sub^ cells (*n* = 3). Cell lysates were stained for SMAD2 total, pSMAD2L and pSMAD2C. β-Actin served as a loading control. **b**–**g** Statistical analyses of Western blot results (**a**). Mean intensities (±SD; *n* = 3) were compared for overall abundance (SMAD2^FL^ + SMAD^ΔE3^; **b**, **d**, **f**) using paired *T*-tests (*p* ≤ 0.05) and SMAD2 splice variants (SMAD2^FL^ vs SMAD^ΔE3^; **c**, **e**, **g**) by RM one-way ANOVA with Sidak’s post hoc test (*p* ≤ 0.05). **h** Western blot of SMAD2 total in A549^WT^ and A549L^sub^ stimulated with inhibitors against CDK1, CDK2 and CDK4/6 (10 µM) or respective controls for 24 h. **i**, **j** SMAD2 splice variant ratios (SMAD2^ΔE3^:SMAD2^FL^) determined by Western blots in NSCLC cell lines, depicted as floating bars (*n* = 3). For statistical comparison, RM one-way ANOVAs with Sidak’s post hoc tests (*p* ≤ 0.05) were performed; CDK1-i., −2-i., −4/6-i Cyclin dependent kinase 1, −2, −4/6 inhibitor, S2FL SMAD2 full-length, S2ΔE3 SMAD2 delta exon3; *p ≤ 0.05; **p ≤ 0.01; ***p ≤ 0.001; ****p ≤ 0.0001.

Antibodies directed against human total SMAD2, pSMAD2L, or pSMAD2C were applied for Western blot. We discovered two bands of SMAD2 in NSCLC cell lines. Based on the molecular weight, we assumed that the lower molecular weight band of total SMAD2 represented the alternatively spliced short variant lacking Exon3 (E3; SMAD2^ΔE3^), which encodes for a 30-amino acid insert within the MH1 domain in full-length SMAD2 (SMAD2^FL^) (Fig. 1c), and was validated by RT-PCR and sequencing (Supplementary Fig. 1M). SMAD2^ΔE3^ is differentially expressed during *X. laevis* embryogenesis [31] and is indispensable for murine embryonic development [29]. However, we observed increased expression of SMAD2 total (SMAD2^FL^ + SMAD2^ΔE3^) in A549L^sub^, which resulted from a significant SMAD2^ΔE3^ gain in cells with absent pSMAD2L, whereas SMAD2^FL^ was comparably expressed in both cell lines (Fig. 3a–c). In A549^WT^, SMAD2^FL^ was the predominant splice variant with a ratio (SMAD2^FL^:SMAD2^ΔE3^) of 14.93 ± 3.91, whereas both SMAD2 splice variants were almost equally expressed with a ratio of 1.04 ± 0.02 in A549L^sub^ lacking pSMAD2L (Fig. 3a–c). The absence of pSMAD2L did not result in disturbance of canonical TGFβ signalling as indicated by equal levels of pSMAD2C [32, 33] (Fig. 3a, d–g). This was further confirmed by TGFβ1 stimulation of both cell lines (Supplementary Fig. 4A).

To analyse whether modulation of pSMAD2L by inhibition of CDK1, CDK2 and CDK4/6 had an effect on short and long splice variant abundance, we performed Western blot analyses of A549^WT^ and A549L^sub^ cells upon stimulation with CDK1, −2, and −4/6 inhibitors (Fig. 3h). No changes were observed for A549^WT^ exposed to inhibitors against CDK1 and CDK2. By contrast, inhibition of CDK4/6, which reduces pSMAD2L in wild-type cells, induced a strong increase of SMAD2^ΔE3^ (Fig. 3h, top). This infers a reverse connection of linker phosphorylation with expression of the short splice variant upon inhibition of the transition from G1/G0 to S phase. In A549L^sub^, SMAD2^FL^ and SMAD2^ΔE3^ protein levels remained unchanged and almost equal by any of the CDK inhibitors, as already observed under unstimulated conditions (Fig. 3h, bottom and I). We then extended the analyses to additional cell types.

### SMAD2^FL^ is predominant but not exclusive in NSCLC cell lines and AECII, whereas SMAD2^ΔE3^ is absent in benign T cells

Both splice variants were expressed with varying ratios in four additional NSCLC cell lines. We compared these results with non-malignant AECII, as they exhibit high cell plasticity and are discussed as possible progenitor cells for NSCLC [34]. Again, SMAD2^FL^ was predominantly expressed compared to SMAD2^ΔE3^ and the SMAD2 splice variant ratio was most similar to A549^WT^ (Fig. 3j; Supplementary Fig. 3A). Concerning phosphorylation, we observed similar pSMAD2L abundance but significantly decreased C-terminal phosphorylation of SMAD2^FL^ in AECII compared to A549^WT^ (Supplementary Fig. 3B, C). In benign T cells, pSMAD2L was only expressed during proliferation, and SMAD2^ΔE3^ was not detected independent of their proliferation status (Supplementary Fig. 1I–L). Consistent with this, pSMAD2L was also present in untreated proliferating JURKAT cells, but in contrast, these cells concomitantly expressed SMAD2^ΔE3^ (Supplementary Fig. 1H).

Our results show that suppressed phosphorylation of SMAD2 within the linker region leads to increased SMAD2^ΔE3^ abundance in A549L^sub^, which is known to exert central functions during embryo development [29, 31]. We next investigated whether additional cellular core processes are modulated by pSMAD2L signalling.

### pSMAD2L is related to developmental processes, cancer-associated pathways and promotes migration

**Fig. 4:**
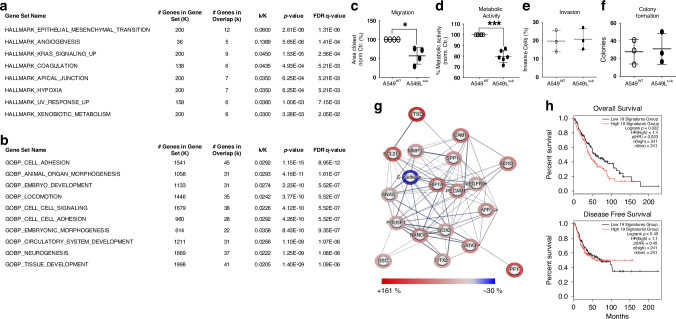
Lack of pSMAD2L results in altered gene expression and affects cellular processes and protein expression in A549 cells. **a**, **b** Differentially expressed genes obtained from transcriptome analyses (moderated *T*-test; *p* ≤ 0.05) in A549^WT^ and A549L^sub^ (*n* = 3) were analysed for gene set enrichment using GSEA MSigDB. Significant terms and corresponding *q*-values (FDR ≤ 0.05) for HALLMARK (H) and GENE ONTOLOGY BIOLOGICAL PROCESS (GOBP) are listed in the tables. **c** The migration ability of A549^WT^ and A549L^sub^ was compared by wound healing assays (*n* = 4). Closed areas after 24 h are expressed as percentages (±SD), A549^WT^ served as the control (100%). Means were compared using a paired *T*-test (*p* ≤ 0.05). **d** Mean percentages (±SD) of metabiloc activity were compared in A549^WT^ and A549L^sub^ cells. Values were compared using a paired *T*-test (*p* ≤ 0.05). **e** Invasive cells passing Matrigel layer after 24 h in a transwell system (*n* = 3) are depicted in percentages (±SD). For comparison, a paired *T*-test (*p* ≤ 0.05) was performed. **f** Mean numbers of colonies (±SD) grown in soft agar for 21 days (*n* = 3) were statistically compared using a paired *T*-test (*p* ≤ 0.05). **g** Network of significantly different protein abundances in A549L^sub^ compared to A549^WT^ (*n* = 3), as determined by multiple *T*-tests (FDR ≤ 0.05), were expressed as percentages and visualised using the STRING database (*string-db.org*). Line thickness indicates data strength (confidence: medium), and halo colour intensities represent the magnitude of change (blue = ↓; red = ↑). **h** Differentially expressed protein signature (F) was analysed for overall survival (OS) and disease-free survival (DFS) using GEPIA-2 (Gene Expression Profiling Interactive Analysis; *gepia2.cancer-pku.cn*) considering LUAD and LUSC. The cutoff value was set to the quartile; *p ≤ 0.05; HR hazard ratio.

We compared transcriptome profiles of unstimulated A549^WT^ and A549L^sub^ to determine pSMAD2L-dependent gene signature followed by over-representation analysis of Hallmark and GOBP gene sets. The pSMAD2L-specific gene signature comprised 351 differentially expressed genes compared to wild-type cells, which encode for cancer-associated processes such as EMT, angiogenesis, and KRAS signalling (Fig. 4a). This was accompanied by significantly enriched GOBP terms such as embryonic development, neurogenesis, as well as tissue and organ morphogenesis. In addition, we obtained significant results for cell-cell signalling, cell (-cell) adhesion and locomotion (Fig. 4b). To verify this, we analysed migration, metabolic activity, invasion, and colony-forming potential of pSMAD2L-deficient cells. Wound healing assays revealed that cells with intact pSMAD2L signalling showed significantly accelerated migration capability and metabolic rate (Fig. 4c, d). By contrast, there was no difference for invasion (Fig. 4e). Similar results were obtained for colonies grown in soft agar (Fig. 4f). Thus, anchorless growth and invasion appear to be pSMAD2L-independent.

Given the evidence that cancer stem-like cells (CSCs) play a central role in inherent and acquired resistance and represent slowly cycling cell population [35, 36], we examined the effects of missing pSMAD2L on cancer- and pluripotency-related protein abundances, as slower proliferation, an increased SMAD2^ΔE3^ level, and GOBP terms related to ontogenesis were enriched in A549L^sub^. Pluripotency-associated proteins such as POU5F1/OCT4, NANOG and SOX2 are expressed in embryonic stem cells (ESCs) during embryogenesis, mediating self-renewal and pluripotency. These stem cell markers are also expressed in NSCLC, enabling resistance to chemotherapy and radiotherapy as well as enhancing malignancy and progression [37–40]. We detected eighteen significantly increased and one decreased protein in cells with absent pSMAD2L (Fig. 4g). Higher expressed proteins included stem cell factors such as POU5F1/OCT4 and NANOG, while E-cadherin was reduced. Forwarding these targets to in silico analysis for survival analysis revealed a significant impact on OS with a worse prognosis for lung cancer patients (Fig. 4h). Co-expression of POU5F1/OCT4 and NANOG promotes malignancy and EMT in LUAD [39], whereas reduced E-cadherin is associated with poor prognosis and metastasis in NSCLC patients [41, 42]. Since TGFβ governs fundamental functions during embryogenesis as well as in adult cells [43, 44] and induces EMT and stemness in lung cancer [45, 46], we next analysed whether phosphorylation of SMAD2L, known to be mediated by non-TGFβ signalling pathways [23, 47], affects canonical TGFβ signalling. Therefore, we stimulated A549^WT^ and A549L^sub^ cells with TGFβ1 and performed Western blots as well as transcriptome analysis.

### pSMAD2L directs TGFβ1-dependent gene expression

**Fig. 5:**
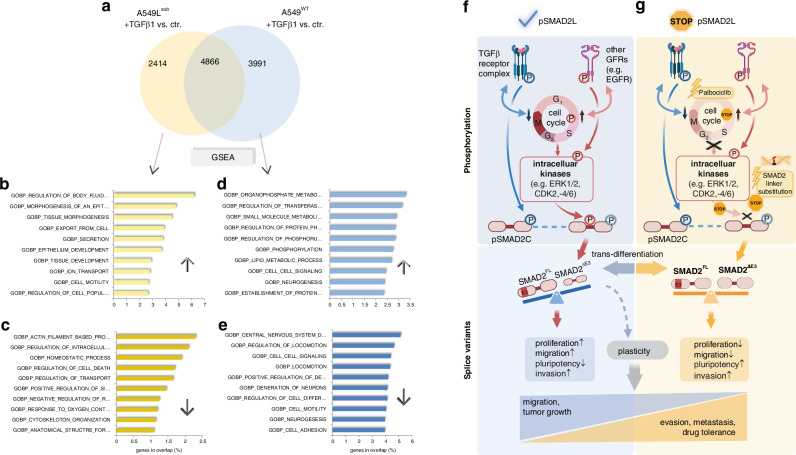
Deprivation of pSMAD2L signalling altered TGFβ1-induced gene expression in A549 cells and contributed to cell plasticity. **a** Prior to transcriptome analysis, A549^WT^ and A549L^sub^ cells were stimulated with human TGFβ1 (5 ng/ml) or respective controls for 48 h (*n* = 3). Differential gene expression between TGFβ1-stimulated and non-stimulated cells was determined by RM one-way ANOVA and Benjamini–Hochberg correction (*p* ≤ 0.05). A Venn diagram was used to identify exclusive. TGFβ1-induced signatures for each cell type. The absence of pSMAD2L expression resulted in 2414 differentially expressed genes in A549L^sub^ and 3,991 genes in A549^WT^. Gene lists were subjected to Gene Set Enrichment Analysis (GSEA) using the Molecular Signature Database (MSigDB). **b**–**e** The 500 most upregulated (↑) and downregulated (↓) TGFβ1-dependent expressed genes (FC ≥ 2) in A549L^sub^ and A549^WT^ cells were analysed using Gene Ontology Biological Process (GOBP; FDR = 0.05). Genes in the overlap of the top ten GOBP terms are shown as percentages in bar plots (*q* ≤ 0.0205). **f** C-terminal TGFβ receptor-mediated phosphorylation of SMAD2 (pSMAD2C) exerts an anti-proliferative effect, whereas phosphorylation of the SMAD2 linker domain (pSMAD2L) promotes proliferation and migration, achieved by non-TGFβ signalling pathways via intracellular kinases (e.g., MAPK, CDK2, CDK4/6). This will result in higher protein abundance of the full-length SMAD2 splice variant (SMAD2^FL^), while short SMAD2 (SMAD2^ΔE3^) is underrepresented. **g** Loss of pSMAD2L signalling by linker-serine substitution or treatment with the CDK4/6 inhibitor Palbociclib causes an increase in short SMAD2, resulting in approximately equal abundance of both SMAD2 splice variants. This leads to lower proliferation and migration, but induction of stem cell factors (e.g. POU5F1/OCT4 and NANOG), whereby invasion capability remained comparable to wild-type cells. Thus, we hypothesised that pSMAD2L enables trans-differentiation of NSCLC and contributes to cancer cell plasticity by transitioning cancer cells from a highly proliferative (EMT/MET = pSMAD2L↑) to a cancer stem cell-like (CSC = pSMAD2L↓) phenotype.

Differentially expressed genes in A549^WT^ and A549L^sub^ were compared using a Venn diagram, to reveal exclusively expressed genes for each cell line (Fig. 5a). Despite SMAD2 linker phosphorylation is not directly transferred by the activated TGFβ receptor complex [23, 47], a considerable amount of genes exclusive to A549L^sub^ cells was found upon TGFβ1 stimulation. We then performed over-representation analysis using GOBP gene sets to elucidate specific biological processes associated with exclusive TGFβ1-signatures in A549L^sub^ and A549^WT^. GOBP terms for epithelial and tissue morphogenetic processes as well as proliferation and motility were significantly enriched among upregulated genes in A549L^sub^ (Fig. 5b). By contrast, GOBP terms for cell death and homoeostatic processes were enriched within downregulated genes (Fig. 5d). These results infer that the absence of pSMAD2L and TGFβ1 stimulation might re-write the cellular differentiation status. In A549^WT^, only GOBP terms for neurogenesis and metabolism as well as locomotion and motility were enriched (Fig. 5e). In summary, our results showed that pSMAD2L is a critical phospho-interface to direct TGFβ1-dependent gene expression, which influences cellular processes playing crucial roles in developmental processes and carcinogenesis (Fig. 5f, g). Therefore, we next examined whether pSMAD2L impacts overall and disease-free survival in NSCLC patients.

### pSMAD2L cell density is enriched in NSCLC and impacts overall survival in a spatially distinct manner

**Fig. 6:**
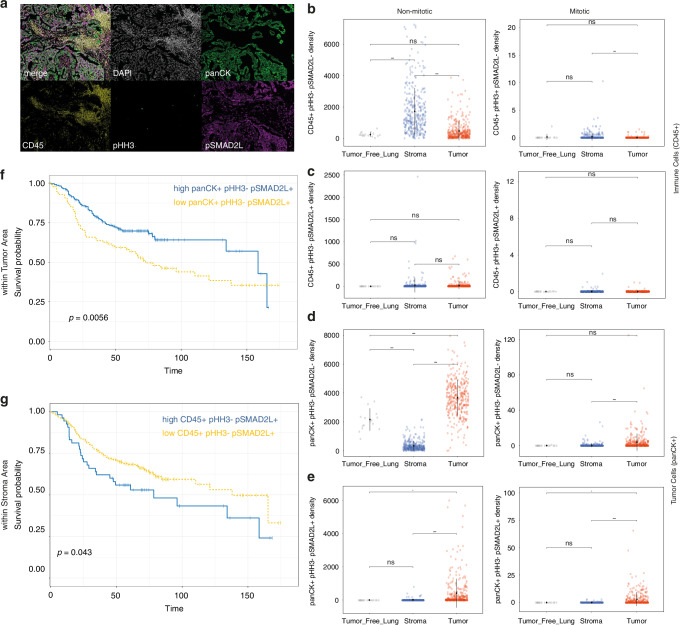
SMAD2 linker phosphorylation is associated with survival in lung cancer. **a** Multiplexed immunofluorescence (mIF) analysis targeting pan-Cytokeratin, CD45, phospho-SMAD2L (pSMAD2L) and phospho-Histone H3 (pHH3) in early-stage non-small cell lung cancer tissues. **b**–**e** Quantification of mIF results for co-expression of pSMAD2L and pHH3 on CD45^+^or panCK^+^ cells within the tumour or stroma areas of NSCLC tissues and tumour-free lung parenchyma. Cell density = mean number of cells for each combination normalised to the total area of each tissue compartment (mm^2^). For Statistical analysis Wilcoxon signed-rank test was used. **f**, **g** Exemplary Kaplan–Meier survival analysis for overall survival (OS) and disease-free survival time (DFS) in months for panCK^+^pHH3^−^pSMAD2L^+^ within tumour compartment and CD45^+^pHH3^−^pSMAD2L^+^ within the stroma compartment. *P*-value computed using Log-Rank test on dichotomised high and low variables.

We investigated the abundance of phospho-SMAD2 linker positive (pSMAD2L^+^) cells in NSCLC and tumour-free tissues by using a 4-plex mIF panel to discriminate epithelial cells (panCK^+^) and immune cells (CD45^+^) as well as their mitotic status, indicated by pHH3 (Fig. 6a). Overall, SMAD2 linker phosphorylation was more common in NSCLC (stroma = 91.81% & tumour = 95.75%) than in tumour-free lung tissues (6.25%). The density of immune cells (CD45^+^_pHH3^−^_pSMAD2L^−^; CD45^+^_pHH3^+^_pSMAD2L^−^) was enriched within the stroma compared to the tumour area, independently of mitosis. No cell density differences were found with respect to CD45^+^_pSMAD2L^+^ cells within the stromal or tumour compartment independent of the pHH3 status (Fig. 6a–c). Non-mitotic epithelial cells were increased in tumour-free lungs and within the tumour compared to stroma (Fig. 6d, left), whereas mitotic epithelial cells were significantly increased in tumour (Fig. 6d, right). pSMAD2L-positive epithelial cells were significantly increased within the tumour compared to stroma and tumour-free lung tissues, independent of the mitotic state (Fig. 6d, e). We found no differences in SMAD2 linker phosphorylation between LUAD and LUSC (Supplementary Fig. 5A–E).

**Fig. 7:**
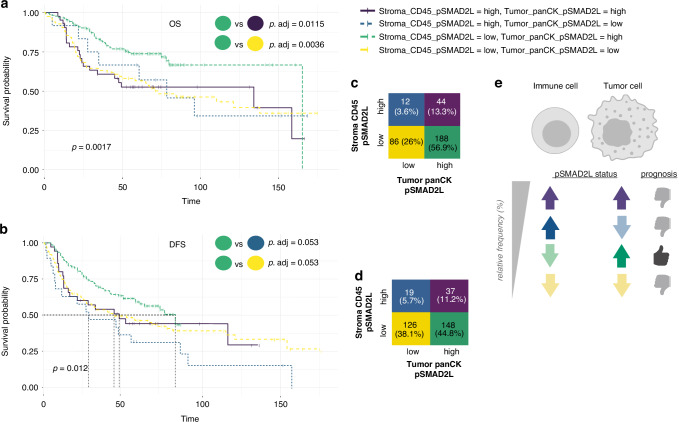
SMAD2 linker phosphorylation in NSCLC affects OS and DFS in a spatially distinct manner. **a**, **b** Categorised NSCLC patients according to pSMAD2L cell density in tumour and stroma areas dichotomised variables (high/low) were analysed to determine the impact on OS and DFS (time = months). **c**, **d** Relative frequency (%) of categorised NSCLC patients with high and low pSMAD2L cell density in tumour and stroma areas. **e** Schematic illustration of the pSMAD2L status in tumour (panCK+) and immune cells (CD45+) as well as the impact on prognosis (created with bioRender.com).

Based on these observations, we next investigated whether pSMAD2L expression had a relevant impact in NSCLC patients on overall survival (OS) and disease-free survival (DFS). For this, we used a feature selection algorithm to identify which variables were impacting OS/DFS and forwarded positive hits to Kaplan–Meier analyses and univariate Cox analyses. Overall, pSMAD2L positivity was associated with a spatially distinct effect, where a low total pSMAD2L level in stroma areas was associated with favourable outcome (HR 0.65, CI 0.41 – 1.04) while a low pSMAD2L level in tumour areas was associated with increased risk (HR 1.71, CI 1.16–2.54) (Supplementary Fig. 5F). If focused on non-mitotic pSMAD2L-positive tumour cells (panCK^+^_pHH3^−^_pSMAD2L^+^), an increased risk of death or relapse (HR 1.68, CI 1.16–2.43 for OS and HR 1.45, CI 1.06–1.98 for DFS) was observed. Conversely, a low level of non-mitotic pSMAD2L-positive immune cells (CD45^+^_pHH3^−^_pSMAD2L^+^) within the stroma parts of cancer tissues was beneficial (OS: HR 0.65, CI 0.42–0.99, DFS: HR 0.69, CI 0.47–0.99) (Fig. 6f, g; Supplementary Fig. 5F). Our results from NSCLC patient tissues indicate that SMAD2 linker phosphorylation is increased within panCK^+^ tumour cells compared to panCK^+^ cells within tumour-free lungs. Furthermore, survival analyses revealed that the effects of SMAD2 linker phosphorylation depend on its cellular context: A low level within immune cells drives beneficial outcome compared to increased risk of death or relapse if happening in tumour cells. Therefore, we next categorised NSCLC patients with high and low pSMAD2L cell density in tumour and stromal areas. We found that patients with high pSMAD2L cell density in the tumour (panCK^+^_pSMAD2L^+^) accompanied by low pSMAD2L cell density in the stroma (CD45^+^_pSMAD2L^+^) had the most favourable prognosis. If one of these pSMAD2L states deviated or occurred in the opposite direction, the prognosis was worse (Fig. 7a, b). Almost 50% of patients exhibited the favourable pSMAD2L status, while the other 50% showed one of the worst pSMAD2L states (Fig. 7c–e).

## Discussion

The TGFβ pathway is a promising target in multiple cancers [48], as Pirfenidone leads to reduced immune evasion and improves immune function while showing strong negative effects in NSCLC [11]. Since inhibition of SMAD2/3 linker phosphorylation is proposed as a novel target for colorectal cancer therapy [13], we investigated the role and presence of pSMAD2L in NSCLC patients, cell lines as well as in primary cells, including AECII and T cells.

### pSMAD2L occurs in a mitosis-dependent manner across germ lines

Overall, pSMAD2L was associated with mitosis in five NSCLC cell lines and proliferating T cells based on increased pSMAD2L expression in G2/M, suggesting a highly conserved mechanism independent of cell lineages. Moreover, mRNA transcription of mitosis-related gene sets was significantly impaired in pSMAD2L-deficient cells, which was functionally confirmed by a slowed proliferation rate and reduced cell subpopulation in the G2/M phase. These results support that pSMAD2L abrogates TGFβ receptor-mediated C-terminal anti-proliferative effects in NSCLC. Similarly, constitutive pSMAD2/3L signalling has also been observed to promote proliferation in melanoma cell lines [49]. In addition, we analysed co-localization of pSMAD2L with pHH3 and PCNT, which are well-established markers for mitosis [50] and centrosomes [51]. Consistently, pSMAD2L is associated with the mitotic apparatus in HeLa cells, and its expression can be modulated by the microtubule polymerisation inhibitor Nocodazole [24]. pHH3 was modulated to a similar extent in the same direction as pSMAD2L, indicating that pHH3 and SMAD2 serine-linker sites might be phosphorylated by the same kinases and fulfil similar functions during mitosis, which remains to be further elucidated. Similarly, further studies are necessary to analyse in detail why pSMAD2L, which is known to function as a transcription factor, occurs centrosome-associated in A549^WT^.

To the best of our knowledge, this is the first study showing that CDK2 and −4/6 are responsible for linker phosphorylation of SMAD2 in NSCLC, suggesting a possible therapeutic approach, e.g., by inhibition of CDK4/6 using Palbociclib, an approved drug for breast cancer [52].

### Alternative splicing of SMAD2 depends on SMAD2 linker phosphorylation and impacts pluripotency-associated proteins

Our results obtained so far imply a possible use of CDK4/6 inhibitors to circumvent pSMAD2L mediated pro-proliferative effects to treat NSCLC. However, upon substitution of the linker sites, we observed induction of the short splice variant SMAD2^ΔE3^, which was associated with pluripotency-related protein regulation in vitro. Elevated SMAD2^ΔE3^ and reduced pSMAD2L levels were also observed upon CDK4/6 inhibition in A549^WT^. Moreover, high pSMAD2L^+^ tumour cell density with a favourable prognosis is somehow questioning the use of Palbociclib.

Protein splice variants are thought to contribute to cancer cell plasticity by mediating epithelial or mesenchymal programs (EMT/MET), which are critical for physiological (embryo development, wound healing) and pathological (cancer, fibrosis) cell states [53]. SMAD2 splice variants are associated with various differentiation processes [30, 54], and equal abundances of both SMAD2 splice variants were observed in *X. laevis* embryos [31]. Notably, SMAD2 splice variants exert non-redundant functions, demonstrated in mouse embryos that developed normally and fertile when *Smad2*^*ΔE3*^ was expressed exclusively in a *Smad3*^−^^/^^−^ genome background, whereas *Smad2*^*FL*^ was dispensable [29]. It is discussed that oncofetal reprogramming in cancer cells, accompanied by reactivation of pluripotency-related proteins, contributes to immune evasion and promotes tumour growth and metastasis [55–57]. Since AECII are able to differentiate into AECI [58], SMAD2^ΔE3^ abundance might reflect their cellular differentiation potential. Consistently, the abundance of SMAD2^ΔE3^ was also related to the differentiation status in T cells. While benign T cells lacked short SMAD2, it was present in malignant T lymphoblasts.

A priori, linker phosphorylation of SMAD2 seems to be responsible for maintaining cellular differentiation and is associated with a favourable prognosis, while the short splice variant is induced upon loss of linker phosphorylation, thereby enhancing pluripotency protein levels and directing NSCLC towards de-differentiation (Fig. 5f, g). Since we extensively showed that induction of the short splice variant of SMAD2 affects pluripotency protein abundance, the use of Palbociclib, a drug mimicking the phenotype of A549L^sub^, would likely result in detrimental effects for the patients.

### SMAD2L is a novel unifying mediator for canonical and non-canonical TGFβ signalling

We observed a gain of alternatively spliced short SMAD2^ΔE3^ in A549L^sub^ cells lacking pSMAD2L, which was expressed almost equally compared to SMAD2^FL^. Upon TGFβ1 stimulation, C-terminal phosphorylation of both SMAD2 splice variants was similarly increased, independent of total SMAD2^FL^ and SMAD2^ΔE3^ levels and the pSMAD2L status, indicating that both are critical for TGFβ1-dependent gene expression.

Given that canonical and non-canonical TGFβ pathways affect chromatin accessibility [59] and that CDK4/6 inhibition induces chromatin remodelling by enhancer activation in breast cancer [52], SMAD2 splice variants and corresponding phospho-isoforms are critical modulators of gene transcription in NSCLC. We showed that TGFβ1-induced gene expression depends on SMAD2L phosphorylation (Fig. 5a–e). This means that canonical signal transduction by TGFβ1 depends on the non-canonical phosphorylated linker site of SMAD2, thereby serving as a unifying element between both sides of the TGFβ pathway.

## Conclusions

Overall, our study reveals that pSMAD2L governs cancer-related cellular processes and redirects canonical TGFβ signalling in vitro. In a therapeutic context, it is conceivable that inhibition of intracellular signalling cascades by drugs such as Sotorasib (KRAS inhibitor) [60, 61] reduce pSMAD2L and lead to a phenotype comparable to A549L^sub^, which is currently under investigation. Our results show that the benefit of reduced proliferation and migration by inhibiting SMAD2 linker phosphorylation would further require counterbalancing of SMAD2^ΔE3^ to prevent induction of pluripotency proteins and de-differentiation.

## Supplementary information

Supplementary Fig. legends

Supplementary Fig. 1

Supplementary Fig. 2

Supplementary Fig. 3

Supplementary Fig. 4

Supplementary Fig. 5

## Data Availability

Transcriptome data are available in the GEO database under GSE277166 and GSE277168.
