# Supplementary material for: SMAD2 linker phosphorylation impacts overall survival, proliferation, TGFβ1-dependent gene expression and pluripotency-related proteins in NSCLC
Source: Br J Cancer. 2025 May 3;133(1):52–65. doi: 10.1038/s41416-025-02970-1 (PMC12238225; doi:10.1038/s41416-025-02970-1)
Supplement: Supplementary file 1 — Supplementary Fig. legends [file 41416_2025_2970_MOESM1_ESM.docx]

**Suppl. Fig 1: Generation of a phospho-SMAD2 linker deficient cell line (A549L^sub^). Analysis of SMAD2 phospho-isoforms and splice variants in benign T cells and malignant T lymphoblasts.** (A) PCR of A549L^sub^ cells cultured from single cell clones (CRISPR/Cas9). Extracted SMAD2 PCR fragment (723 bp) was restricted with *HaeII* (n=1). Upon successful cloning, bands of 465 bp and 253 bp were expected. (B) SMAD2 PCR fragment of A549L^sub^ cells (n=1) was sequenced and showed successful template insertion and replacement of the SMAD2 linker serine triplet to alanine (yellow). (C) pSMAD2L abundance in A59L^sub^ and A549^WT^ cell lysates (n=3) was examined by Western blot using different primary antibodies. (D) T cells from whole blood samples of healthy donors (n=4) were activated by CD3/CD28-beads and IL2 (TC^+^), non-activated T cells (TC^-^) served as control. To verify sufficient proliferation upon T cell activation, cells were stained with DAPI followed by cell cycle analysis using flow cytometry. Paired T-tests (p≤0.05) were performed for statistical comparison. (E) Representative Western blots of SMAD2 total and pSMAD2C abundance in TC^+^ and TC^-^ (n=3). β-Actin served as loading control. (F+G) Paired T-tests (p≤0.05) were performed to compare Western blot results for SMAD2 total and pSMAD2C abundance in TC^+^ and TC^-^. (H) Representative Western blots of SMAD2 total and pSMAD2L abundance in JURKAT cells (n=3). β-Actin served as loading control. (I) Products of RT-PCR using SMAD2 exon 3 spanning primers, either full length (362 bp) or short *SMAD2* (272 bp) were extracted from the agarose gel and subjected to Sanger sequencing. Sequences obtained for A549^WT^ and A549L^sub^ were submitted to NCBI BLASTn. Query coverage (QC) values for *SMAD2^FL^* and *SMAD2^ΔE3^* transcripts are given as percentages in the table; notes: SMAD2 total = SMAD2 regardless of phosphorylation; pSMAD2C = phospho-SMAD2 C-terminus; pSMAD2L = phospho-SMAD2 linker; S2FL/SMAD2^FL^ = SMAD2 ‘full-length’; S2ΔE3/SMAD2^ΔE3^ = SMAD2 delta exon 3; * = p≤0.05; ** = p≤0.01.

**Suppl. Fig 2: Effects of CDK inhibitors on cell cycle, pSMAD2L and pHH3 expression in NSCLC cell lines (controls).** (A-C) Five NSCLC cell lines were stimulated with 10 µM CDK1-, CDK2- and CDK4/6-inhibitors (CDK1-i.; CDK2-i.; CDK4/6-i.) or cultured in medium or corresponding control conditions for 24 hours. Cell cycle analysis was performed by flow cytometry using FCS express multicycle function via DAPI. Mean values of relative cell counts (%; ±SD) in G0/1, G2/M and S are depicted. Stimulations were compared with their respective controls using RM one-way ANOVA (p≤0.05). (D-M) Flow cytometry analysis of A549^WT^ cells cultured under control conditions. In addition to DAPI, cells were stained for pSMAD2L (AF488) and pHH3 (AF647); notes: ** = p≤0.01; *** = p≤0.001; **** = p ≤ 0.0001.

**Suppl. Fig 3: SMAD2 and phospho-isoform expression in NSCLCL cell lines and AECII. (A)** Depicted are the means (±SD) of both SMAD2 splice variant (SMAD2^FL^ and SMAD2^ΔE3^) intensities in five NSCLC cell lines and AECII obtained from tumor-free lung tissue (n=3). Cell lysates were stained for SMAD2 total and compared by RM-one way ANOVA (p≤0.05) (B+C) Western blot results for SMAD2 total and phospho-isoforms in A549^WT^ and AECII (B). Mean intensities (±SD; n=3) for SMAD2 total, pSMAD2L, and pSMAD2C considering SMAD2^FL^ and SMAD2^ΔE3^ in A549^WT^ and AECII are depicted. Pairwise comparison was performed using RM-one way ANOVA and Sidak’s posthoc test (p≤0.05); notes: S2/SMAD2FL = SMAD2 full-length; S2/SMAD2ΔE3 = SMAD2 delta exon 3; * = p≤0.05; ** = p≤0.01; *** = p≤0.001.

**Suppl. Fig 4: Western blot analysis of TGFβ1 stimulated A549^WT^ and A549L^sub^ cells.** (A) Western blot analysis of A549^WT^ and A549L^sub^ cells lysates upon TGFβ1 stimulation or respective controls for 48 hours (n=3). Cell lysates were stained for SMAD2 total, pSMAD2L and pSMAD2C. β-Actin served as loading control; notes: S2FL = SMAD2 full-length; S2ΔE3 = SMAD2 delta Exon 3; pSMAD2L = phospho-SMAD2 linker; pSMAD2C = phospho-SMAD2 C-terminus.

**Suppl. Fig 5: SMAD2-linker phosphorylation events are independent of the histological subtype in NSCLC but impact OS and DFS in NSCLC patients.** Analysis of total pSMAD2L density (A), panCK+pHH3-pSMAD2L+ density (B), panCK+pHH3+pSMAD2L+ density (C), CD45+pHH3-pSMAD2L+ density (D) and CD45+pHH3+pSMAD2L+ density (E) within tumor areas of lung adenocarcinomas (LUAD) or lung squamous cell carcinomas (LUSC). Statistical analyses using two-sided T test. Cell density = cells/mm^2^. (F) Results from Cox Univariate Regression Analysis of dichotomized variables (high/low) for overall and disease-free survival months from mIF data with respect to their spatial location within the tumor tissues. HR= hazard ratio, CI95% = 95% confidence interval, p val = p value for univariate results.
