# Supplementary figures and images for: SMAD2 linker phosphorylation impacts overall survival, proliferation, TGFβ1-dependent gene expression and pluripotency-related proteins in NSCLC
Source: Br J Cancer. 2025 May 3;133(1):52–65. doi: 10.1038/s41416-025-02970-1 (PMC12238225; doi:10.1038/s41416-025-02970-1)

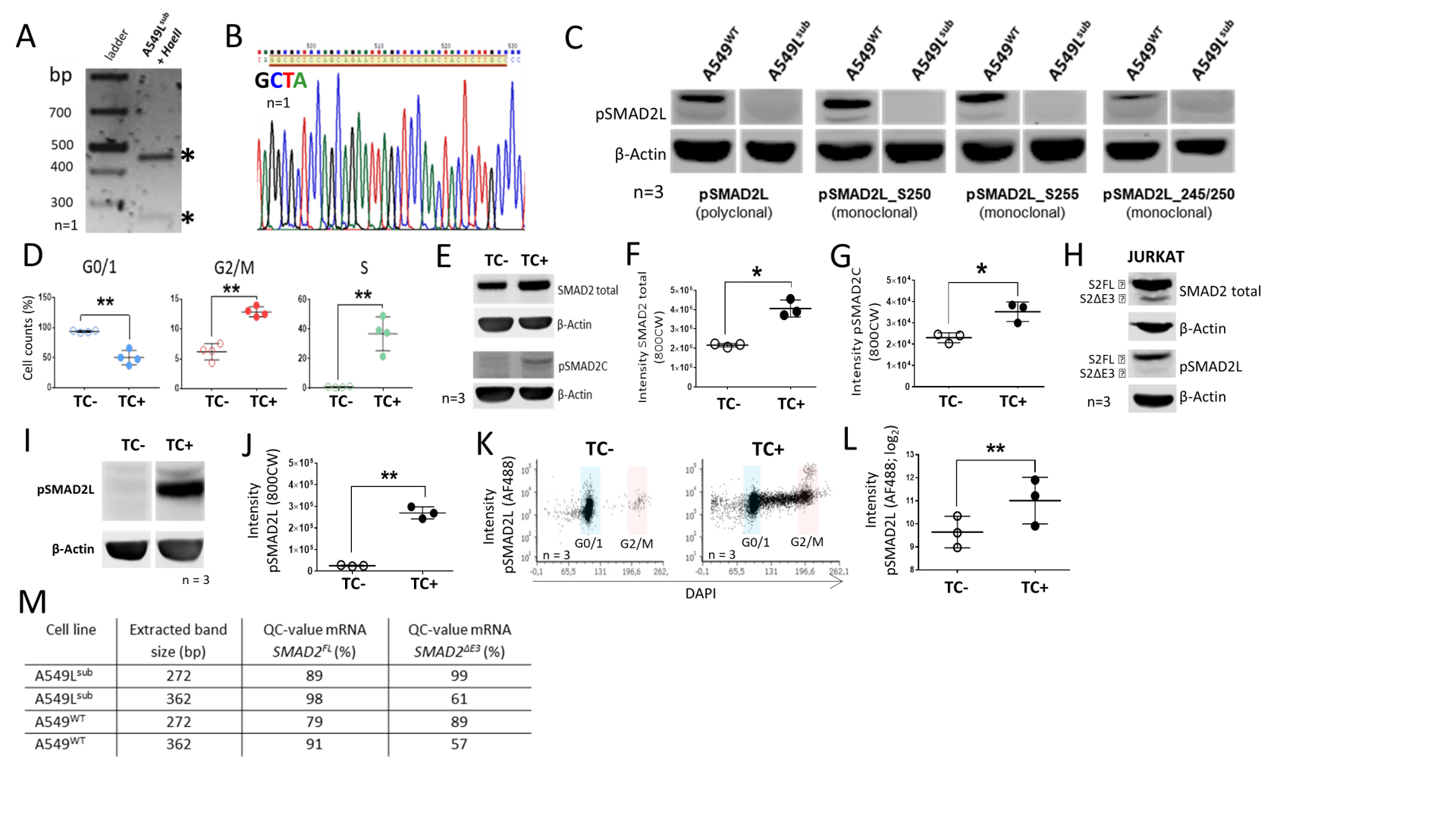

Supplement: Supplementary file 2 — Supplementary Fig. 1 [file 41416_2025_2970_MOESM2_ESM.tif]

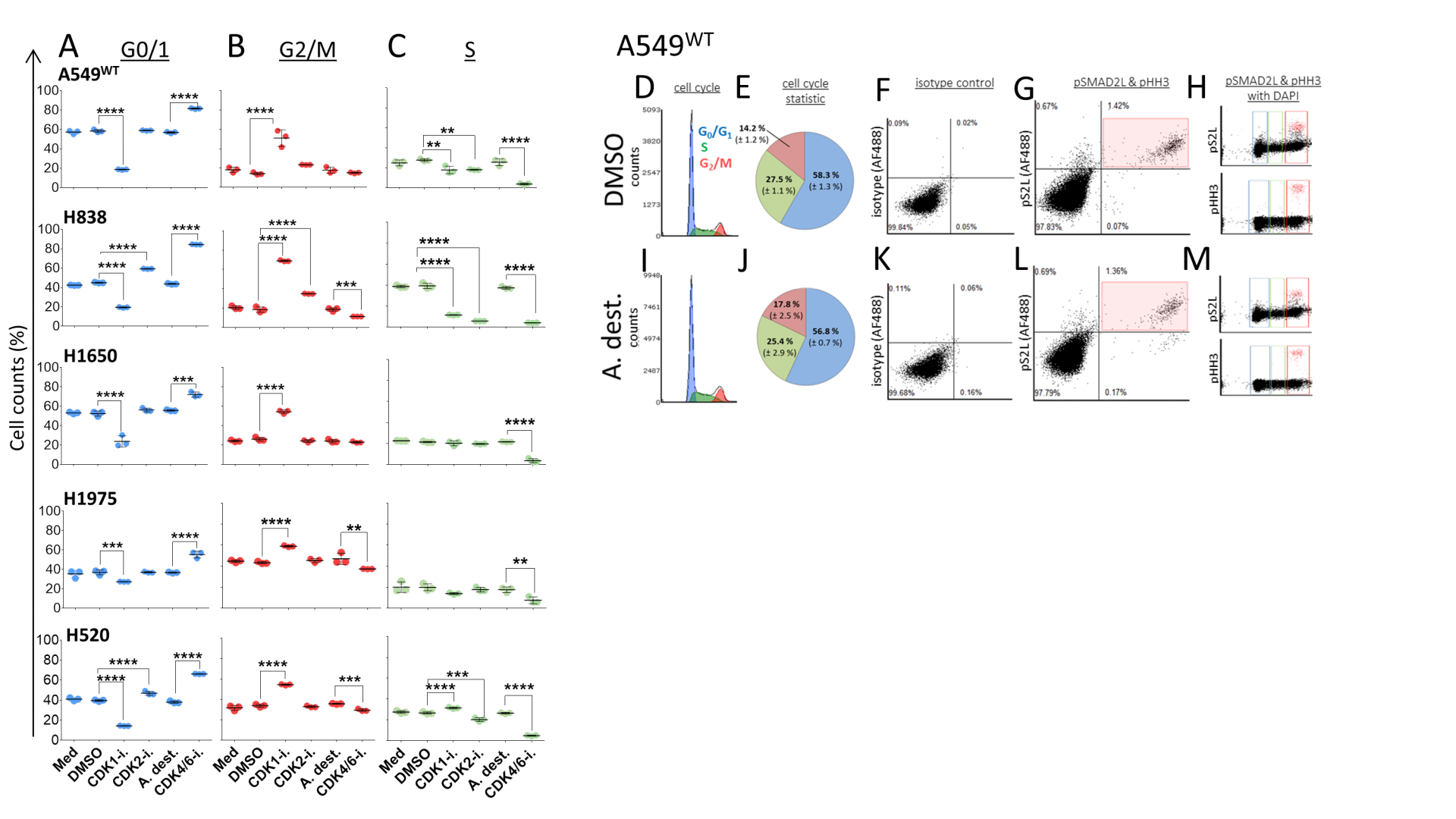

Supplement: Supplementary file 3 — Supplementary Fig. 2 [file 41416_2025_2970_MOESM3_ESM.tif]

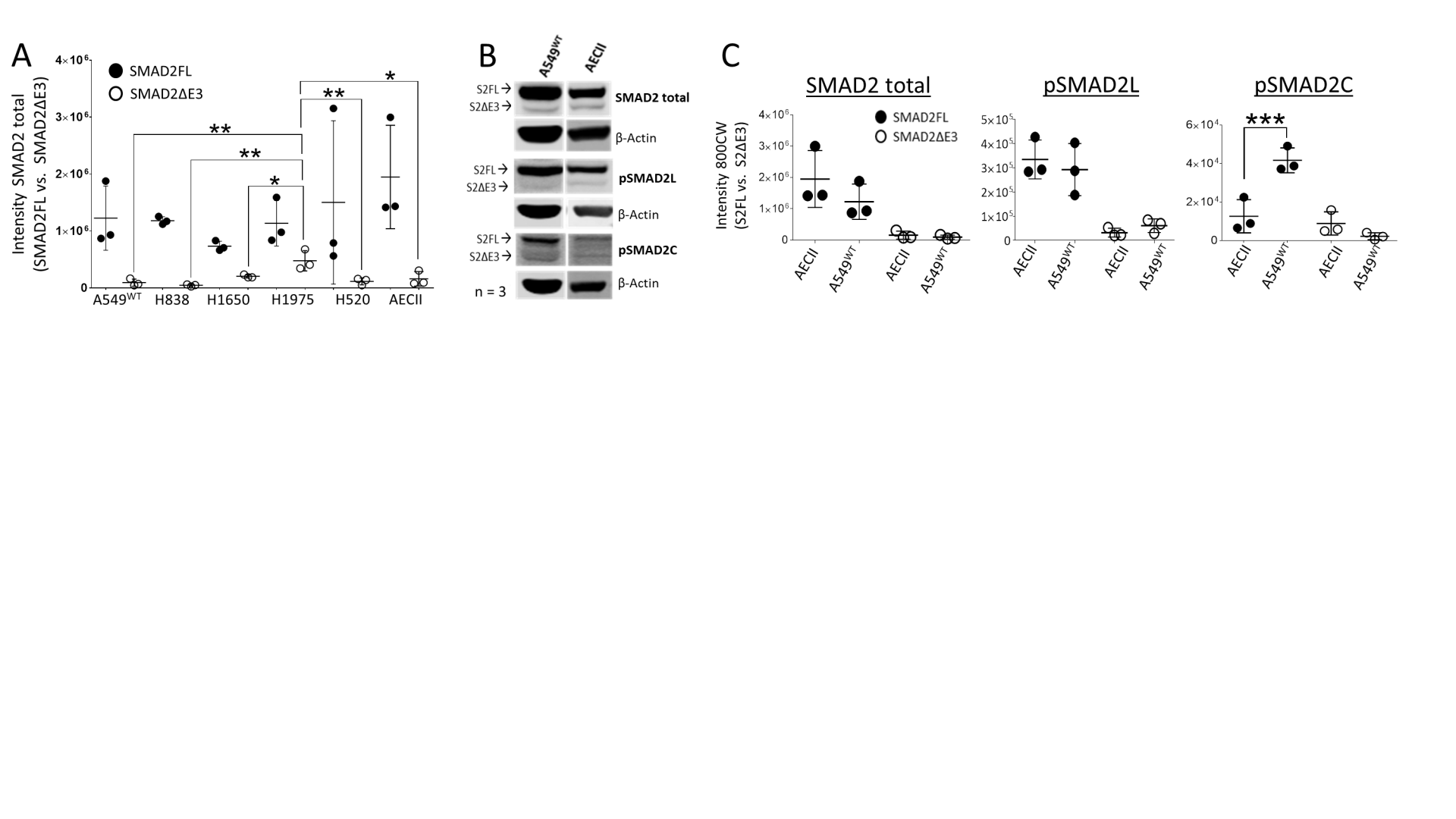

Supplement: Supplementary file 4 — Supplementary Fig. 3 [file 41416_2025_2970_MOESM4_ESM.tif]

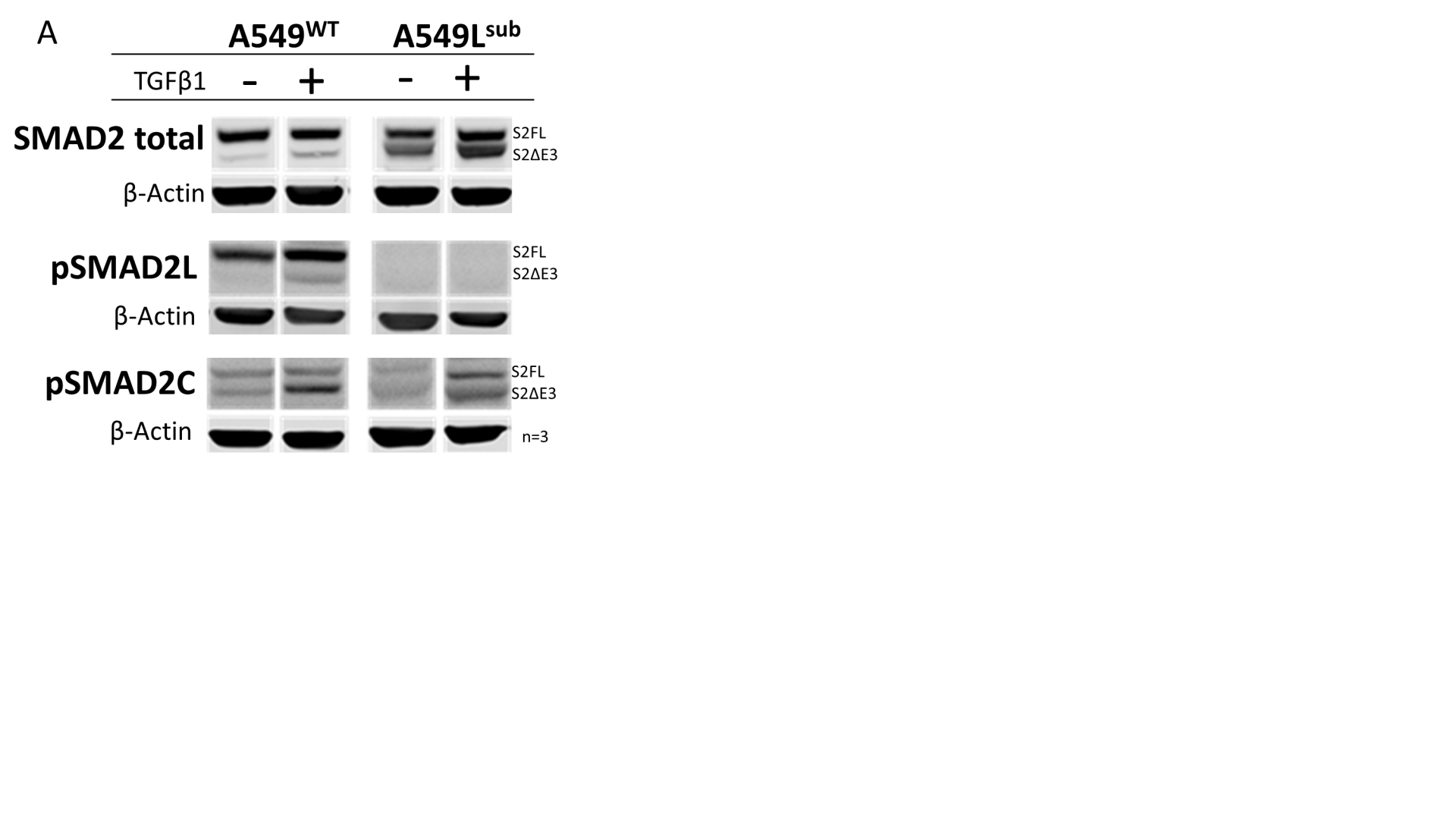

Supplement: Supplementary file 5 — Supplementary Fig. 4 [file 41416_2025_2970_MOESM5_ESM.tif]

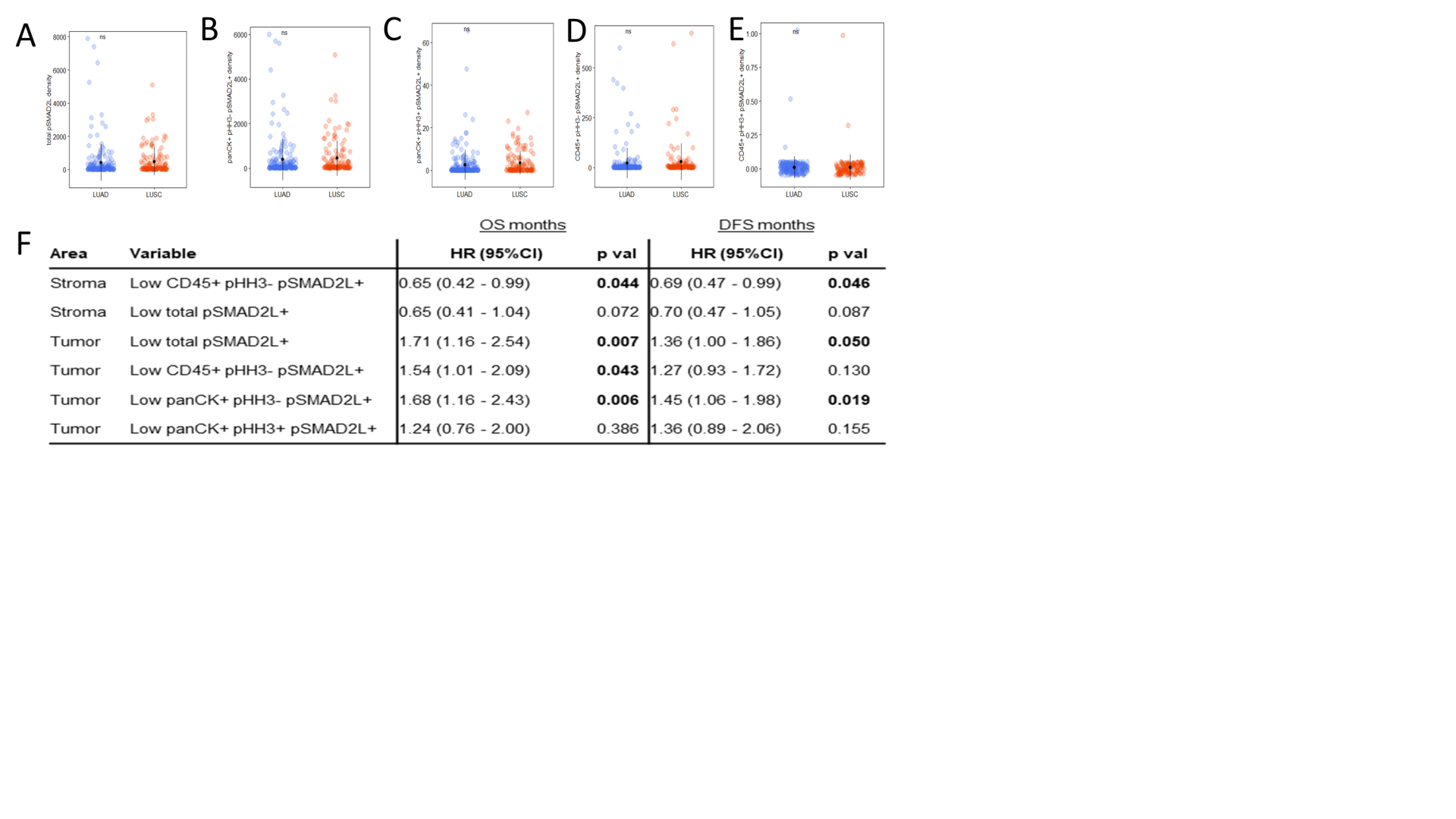

Supplement: Supplementary file 6 — Supplementary Fig. 5 [file 41416_2025_2970_MOESM6_ESM.tif]
